# Normal 3T MR Anatomy of the Prostate Gland and Surrounding Structures

**DOI:** 10.1155/2019/3040859

**Published:** 2019-05-28

**Authors:** K. Sklinda, M. Frączek, B. Mruk, J. Walecki

**Affiliations:** ^1^MD PhD, Dpt. of Radiology, Medical Center of Postgraduate Education, CSK MSWiA, Woloska 137, 02-507 Warsaw, Poland; ^2^MD, Dpt. of Radiology, Medical Center of Postgraduate Education, CSK MSWiA, Woloska 137, 02-507 Warsaw, Poland

## Abstract

Development on new fast MRI scanners resulted in rising number of prostate examinations. High-spatial resolution of MRI examinations performed on 3T scanners allows recognition of very fine anatomical structures previously not demarcated on performed scans. We present current status of MR imaging in the context of recognition of most important anatomical structures.

## 1. Introduction

Aging of the society together with growing consciousness of the role of early detection of oncologic diseases leads to globally occurring rise in number of detected cases of prostate cancer. Widely used transrectal sonography of the prostate gland despite additional support of contrast media and elastography does not provide sufficient sensitivity or specificity of prostate cancer detection. Therefore, in 2012, International European Committee of Urologists and Uroradiologists came up with a set of guidelines regarding magnetic resonance imaging of the prostate gland in order to provide better diagnostic approach, known as PI-RADS-Prostate Imaging, Reporting and Data Set. Shortly after implementation of PI-RADS, multicenter studies have started. On one hand, they have proven its value and, on the other hand, have depicted its weaknesses. Therefore, in 2014, the new version of guidelines, namely, PI-RADS version 2 was presented. It has been prepared in collaboration of American College of Radiology and European Society of Uroradiology and published as a free access online document in 2015. Magnetic resonance imaging of the prostate gland is relatively new and dynamically developing diagnostic tool of urogenital radiology. Expertise in anatomy of the prostate gland and adjacent structures is essential in order to apply PI-RADS v2. Therefore, authors consider the presentation of this subject particularly important. We will also briefly present benign prostate hypertrophy (BPH) which is the most common condition of the prostate, occurring in most patients over 50 years of age.

## 2. Imaging Protocol

According to PI-RADS v2, T1W and T2W sequences should be obtained for all prostate mpMR exams [1]. T1W images are used primarily to determine the presence of hemorrhage within the prostate and seminal vesicles and to delineate the outline of the gland. T2W images are used to discern prostatic zonal anatomy, to assess abnormalities within the gland, and to evaluate for seminal vesicle invasion, EPE, and nodal involvement. These are of utmost importance when it comes to define the anatomy of the scanned region. Multiplanar (axial, coronal, and sagittal) T2W images are usually obtained with fast-spin- echo (FSE) or turbo-spin-echo (TSE) sequences. Recommended slice thickness is 3 mm with no gap. Field of view (FOV) should encompass the entire prostate gland and seminal vesicles, and it generally ranges between 12 and 20 cm.

Diffusion-weighted imaging (DWI) which reflects the random motion of water molecules and is a key component of the prostate mpMRI exam, but it must be correlated with T2W images as it very poorly reflects anatomy. DCE should be included in all prostate mpMRI exams, and it should always be closely inspected for focal early enhancement. If found, the corresponding T2W and DWI images should be carefully interrogated for a corresponding abnormality.

## 3. Basic Anatomic Landmarks

**Figure 1:**
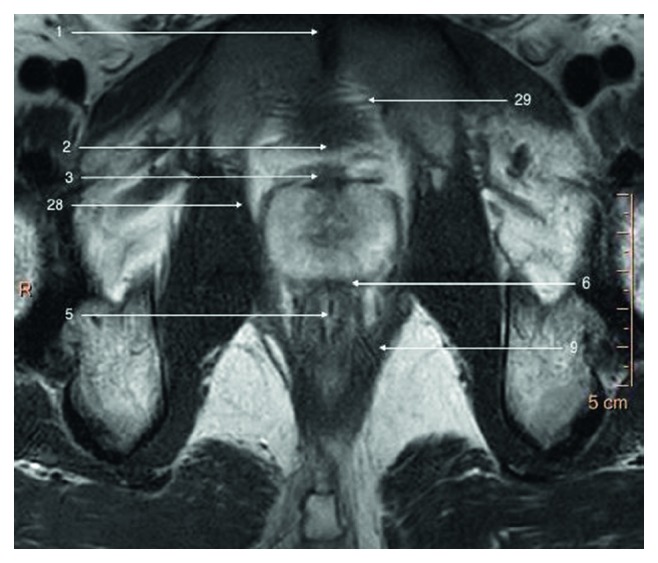
T2-weighted axial image of a prostate gland and surrounding structures.

**Figure 2:**
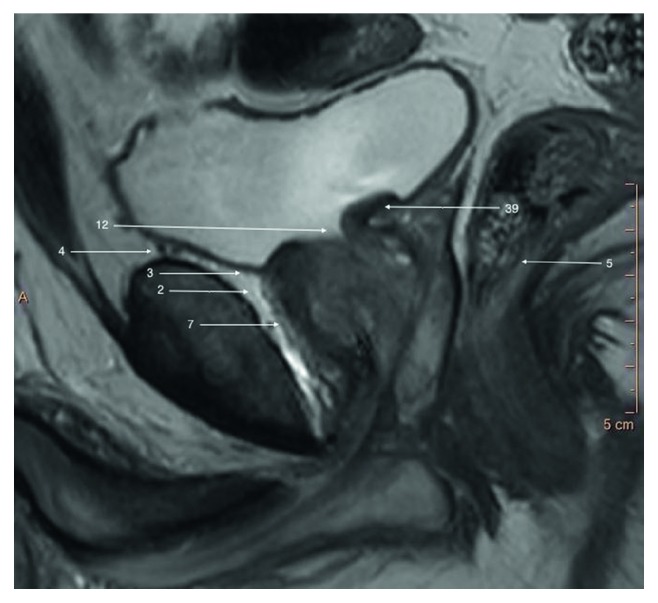
T2-weighted sagittal image of a prostate gland and surrounding structures.

**Figure 3:**
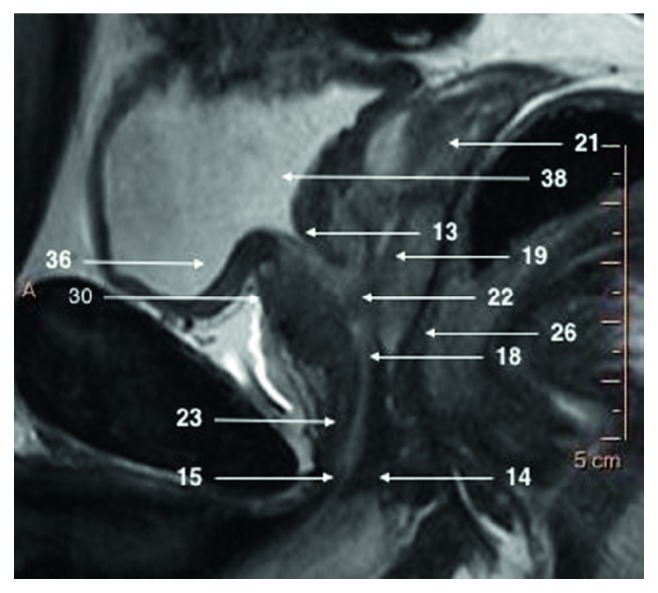
T2-weighted sagittal image focused on a prostate gland.

The prostate gland is nonpaired partly glandular and partly fibromuscular structure located deep in lesser pelvis, surrounding the proximal part of the male urethra and the ejaculatory ducts (Figures 1–3) [2]. The pubic symphisis (Structure 1), retropubic space (cave of Retzius) (Structure 2), puboprostatic ligaments (Structure 3), and vesical venous plexus (Structure 4) can be appreciated anteriorly from the prostate gland. Posteriorly, from the prostate gland, the rectal ampula (Structure 5), Denonvilliers fascia (Structure 6), prostatic venous plexus (Santorini venous plexus) (Structure 7), and prostatic nervous plexus (Structure 8) can be found. Symmetrically on, both lateral sides, the levator ani muscles (Structure 9), parietal and visceral layers of pelvic fascia (Structure 10), and neurovascular triangles occur. The T2W scans in three perpendicular planes are most valuable to perform the anatomical evaluation. Superiorly from the prostate gland, the neck of the urinary bladder (Structure 12) and the internal urethral sphincter (sphincter vesicea) (Structure 13) are seen. Inferiorly from the prostate gland are located the urogenital diaphragm (Structure 14) and the external urethral sphincter (sphincter urethrea) (Structure 15).

## 4. Prostate Gland on MRI

**Figure 4:**
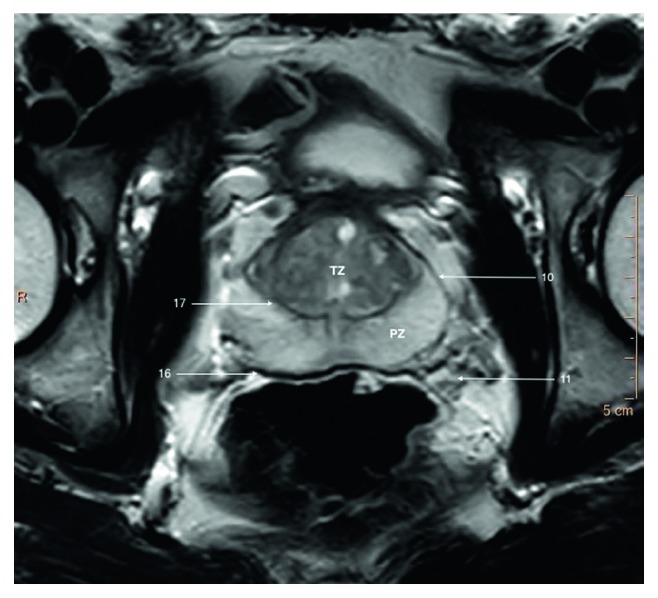
T2-weighted axial image of a prostate gland of a young patient showing clear zonal demarcation.

However, histologically the prostate gland is not surrounded with a capsule, there is a condensation of pelvic fascia (Structure 16) separated from the prostate by the prostatic venous plexus [2, 3]. It can be distinguished on T2-weighted images as a thin hypointense line on the outskirts of the peripheral zone (Figures 3 and 4).

Apart from that, there is a prostatic pseudocapsule (Structure 17) or a surgical capsule to be seen. It is a thin, dark rim on T2W images which occurs due to compression of the prostatic tissue between transition and peripheral zone.

Prostate shape is sometimes compared to sweet chestnut—it is a cone shaped gland, flattened from front to back. From superior to inferior, the prostate is divided into base, midgland, and apex (Figure 3).

Base is the largest part of the gland. Its surface aims superiorly and slightly posteriorly adjoining neck of the urinary bladder. The prostatic part of the urethra (Structure 18) enters the prostate gland here and muscle bands from the bladder wall penetrate fibromuscular capsule of the prostate creating strong connection between the neck of the bladder and anterior part of the base of the prostate gland. Posterior part of the prostatic base has a deep, transverse cleft where the ejauculatory ducts (Structure 19) formed by vas deferens (Structure 20) and seminal vesicles (Structure 21) enter.

Midgland is middle 1/3rd of the prostate gland. It includes the verumontanum (Structure 22) in the midprostatic urethra. The verumontanum (also called seminal colliculus) is distinctive elevation of urothelium with orifaces of the prostatic ducts, ejaculatory duct, and prostatic utricle.

Apex aims inferiorly and slightly anteriorly and adjoins directly deep perineal pouch and even penetrates its muscular tissue. The apex of the prostate (Structure 23) is in close relation with the bulb of penis (Structure 24) and the bulbourethal glands (Structure 25) (Figure 3).

## 5. Zonal Anatomy

Zonal anatomy introduced by McNeal divides prostate into four zones [3]: peripheral zone (PZ), central zone (CZ), transition zone (TZ), and anterior fibromuscular zone (or stroma). Zonal anatomy of the prostate gland is best appreciated on T2-weighted images (Figure 4).

Peripheral zone is the largest zone of the prostate gland and accounts for 70% of the gland in young men. The majority of prostate cancers (70–75%) originate from peripheral zone.

It comprises the posterior aspect of midgland and most of apex of the prostate. On T2W images, the normal peripheral zone can be easily distinguished from central gland by its homogeneously hyperintense signal, whereas the central gland tissue is typically hypointense or isointense compared to skeletal muscle.

Peripheral zone is separated posteriorly from the anterior wall of the rectum (Structure 26) and middle rectal vessels by a thin capsule. It is a part of the gland located closest to rectum and can be felt during DRE.

Bilaterally peripheral zone abuts neurovascular bundles (Structure 11) which course anteriomedially entering prostate at 5 and 7 o'clock.

**Figure 5:**
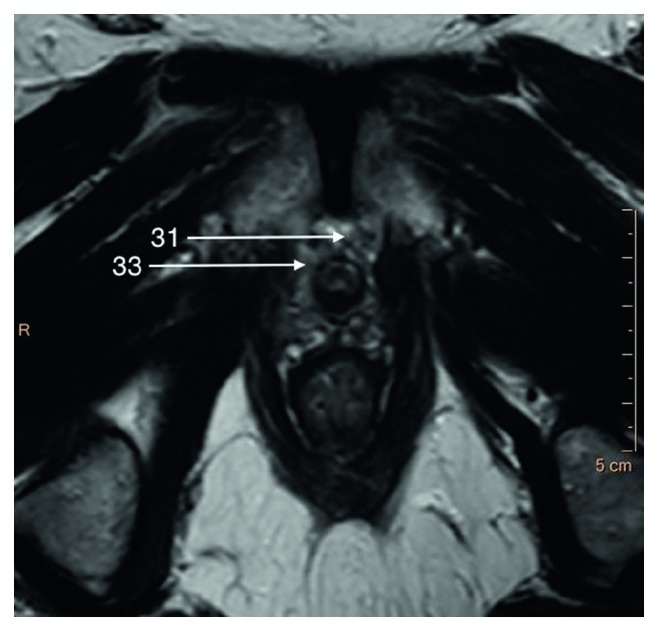
T2-weighted axial image of an apical part of the prostate gland.

**Figure 6:**
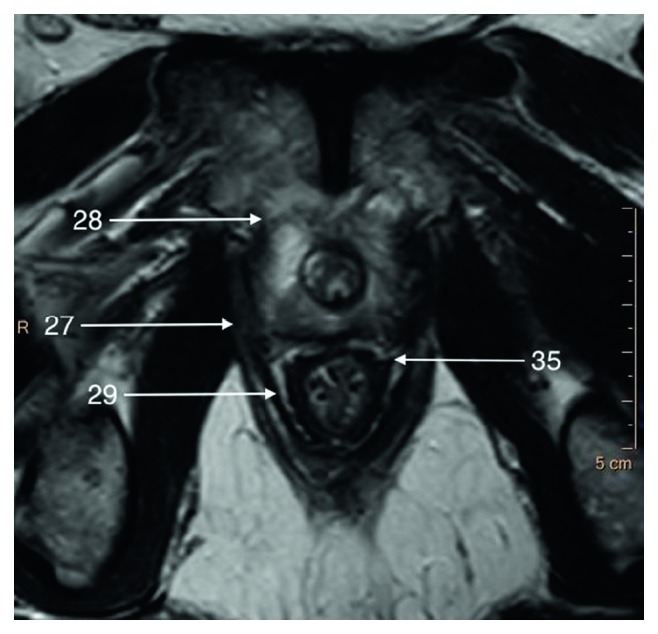
T2-weighted axial image of a border between apical and middle parts of the prostate gland.

In the apex, peripheral zone adjoins laterally and inferiorly the pubococcygeus muscles (Structure 27), anteriorly puboprostatic (levator prostate) muscles (Structure 28), and inferiorly puboanalis muscles (Structure 29) which are parts of levator ani muscle (Structure 9) (Figures 5 and 6).

### 5.1. Central Gland

The transition and central zones of the prostate have similar signal intensity on MRI and therefore cannot be resolved at MR imaging. They are collectively referred as the central gland.

### 5.2. Transition Zone

The transition zone is the innermost section of the prostate and forms 5% of the gland in young adults. It consists of two small lobules and surrounds urethra superiorly to verumontanum—the posterior part of urethra at base and anteriolateral part at midgland.

The transition zone abuts anteriorly anterior fibromuscular stroma, posteriorly central zone at the base, and peripheral zone at the midgland and apex.

### 5.3. Central Zone

The central zone is a wedge-shaped structure located at the base of the prostate between the peripheral and transition zones surrounding the ejaculatory ducts and narrowing to an apex at the verumontanum. It comprises up to 25% of the glandular tissue.

### 5.4. Anterior Fibromuscular Stroma

It is a thick sheath of tissue contiguous with detrusor vesicae (Structure 30) which forms anterolateral border of glandular prostate. It does not contain glandular tissue and is thought to be without importance for prostatic function and pathology. At the base of the prostate, it contacts urethra anteriorly. Pubuprostatic ligament connects AFMS to the inferior edge of pubic symphisis.

On MRI imaging in T2 sequence, the anterior fibromuscular stroma is hypointense due to a lack of glandular tissue.

## 6. Important Structures Assessed during Magnetic Resonance Imaging

The pubovesical/puboprostatic ligaments (Structure 3) (PVLs/PPLs) are paired fibrous structures extending from the outer longitudinal bladder muscle to the distal part of the posterior part of the pubic symphysis (Structure 1) adjacent to the anterior margin of internal urethral sphincter (Structure 13) creating so-called detrusor apron [4]. These ligaments play a significant role in the suspensory system of the continence mechanism. Their appreciation is difficult in cases of patients suffering from BPH.

The fascias which are important for radical prostatectomy as they represent surgical dissection planes partially recognizable on MRI. The nomenclature referring to pelvic fascias is variable since some authors distinguish parietal and visceral fascia and others do not naming it endopelvic fascia (Structure 31), which we follow in this paper (Figure 5). The endopelvic fascia covers the rectum, urinary bladder, and prostate fusing with the anterior fibromuscular stroma of the prostate, levator ani muscles (Structure 9), and the pelvic sidewalls. It propagates from the PVLs/PPLs to the ischial spine (Structure 32) (Figure 5).

**Figure 7:**
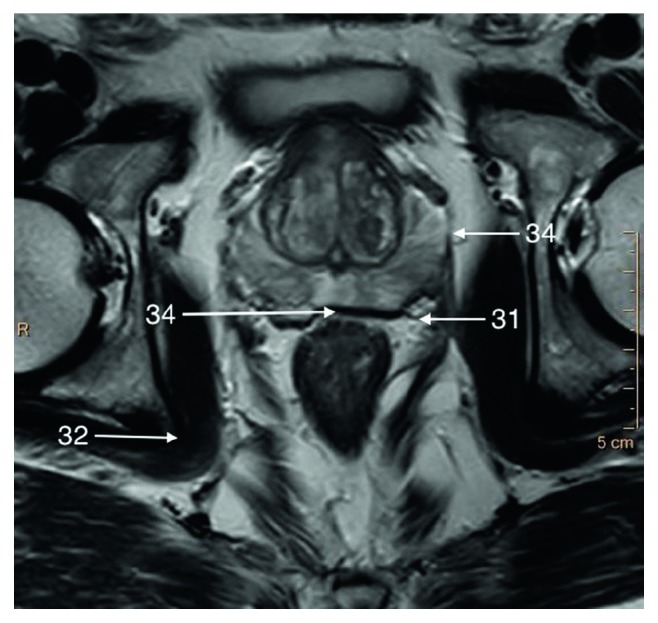
T2-weighted axial image of a middle part of the prostate gland.

The periprostatic fascia (PPF) is a multilayer mixed collagenous-adipose structure which covers lateral surfaces of the prostate gland externally to the prostate capsule. For the surgeon, the levator ani fascia (Structure 33) laying outermost and prostatic fascia (Structure 34) covering the prostate capsule merge into PPF with a neurovascular bundle (NVB) (Structure 11) passing usually between them (Figures 5 and 7). Dorsally to the NVB, the levator ani fascia turns into pararectal fascia (Structure 35), which divides rectum from the levator ani muscle (Figure 6). The prostatic fascia extends anteromedially to the NVB covering the prostate capsule. The posterior coverage of the prostate and the seminal vesicles is called Denonvillers' fascia (also rectoprostatic fascia or posterior prostatic fascia) (Structure 6). It is connected to the prostatic capsule in the midline and ends at the prostatourethral junction.

The neurovascular bundle (NVB) (Structure 11) as mentioned earlier is situated laterally and in the posterolateral angle of the prostate and seminal vesicles (Structure 21) as a bundle or spread out within adipose-connective tissue between layers of prostatic (Structure 34) and levator ani (Structure 33) fascia (Figures 5 and 7). Its complicated and not fully described character is only poorly seen on MR images; therefore, it will not be described in detail in this paper.

The prostatic capsule (Structure 16) is in fact a layer of mainly smooth muscular fascicles fused with the stromal tissue dorsally and laterally. In front, it is fused with the anterior fibromuscular stroma and detrusor apron (Structure 3), at the apex with the external uretral sphincter (Structure 15) and at the base with the bladder detrusor (Structure 30), and therefore, it cannot be clearly delineated there on MR imaging.

The urinary bladder (Structure 36) is located superiorly to the prostate. It is basically divided to base located superiorly and neck located inferiorly.

**Figure 8:**
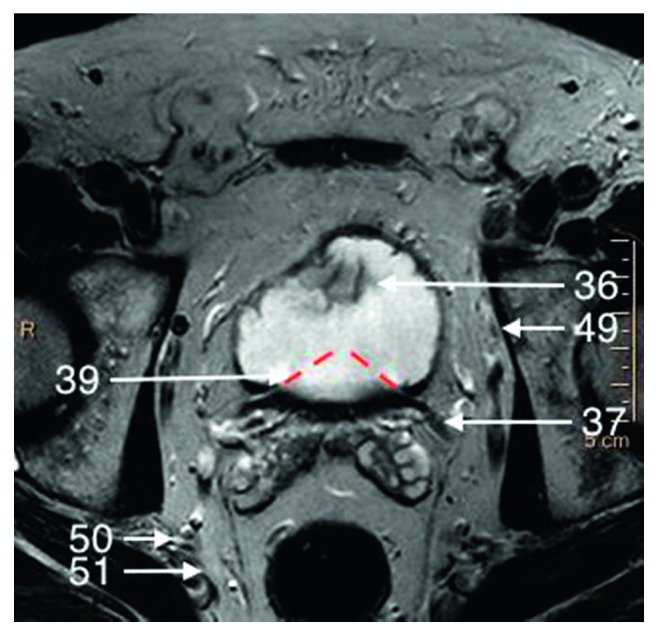
T2-weighted axial image of a basal part of the prostate gland.

The base of the urinary bladder adjoins laterally and posteriorly ureters (Structure 37) with the orifaces of ureters penetrating obliquely wall of the bladder in the posteriolateral angles of trigone of the bladder (Structure 38) (Figures 7 and 8). The neck of the bladder (Structure 12) thins anteriorly to become internal orifice of urethra. Posterior wall of bladder touches seminal vesicles (Structure 21), ampulae of vas deferens (Structure 20), and bladder venous plexus (Structure 4) (Figures 2 and 3).

Prostatic urethra (Structure 18) is 3-4 cm long. It descends from base to apex of the prostate nearly vertically forming anteriorly concave curve. Highest part of the urethra called intramular part is subtended anteriorly by the internal urethral sphincter (Structure 13) and the prostate laterally and posteriorly. In the posterior aspect of the urethra, in extension of vesical uvula lies urethal crest with the seminal colliculus (Structure 22), where ejaculatory ducts (Structure 19) and prostatic duct open (Figures 3 and 7).

After leaving prostate, the membranaceus part of urethra begins where it passes through muscles of urogenital diaphragm (Structure 14), external urethral sphincter (Structure 15), and deep and superficial transverse perineal muscles.

External urethral sphincter (Structure 15) is an important anatomical structure while planning operation on prostate as it needs to be spared during radical prostatectomy in order to maintain continence. It is found caudal to the prostate apex. It is independent from the muscles of the pelvic floor although it is in close relationship to them. It is a double-layered striated-smooth muscle innervated by autonomic branches of the pelvic plexus which enter the sphincter posterolaterally from both sides. Its fibers are continuous with membranaceus part of urethra. The external urethral sphincter forms embryologically as a separate muscle but later in life accretes with deep transverse perineal muscle and eventually they become unseparable ((Figure 3).

Vesicoprostatic muscle (Structure 39) is located between the bladder neck (Structure 12), the insertion of the seminal vesicles (Structure 21), and the ampullae of the vasa defferentia (Structure 20) (Figures 2 and 8).

Levator prostatae (or urethrae) (Structure 28) is the anterior part of the levator ani (Structure 9)—the innermost muscle of pelvic floor musculature (Figure 6). Together with puborectal muscle (Structure 29), they play an active role in abrupt stop of micturition. Superficial transverse perineal muscle lies inferiorly to external urethral sphincter (Structure 15) and deep transverse perineal muscles.

The seminal vesicles (Structure 21) are multicystic structures located above the posterior part of prostatic base—the central zone and adjoin the urinary bladder (Structure 36) [4]. The medial edge adjoins ampulla of vas deferens (Structure 20). Lateral edge abuts the wall of lesser pelvis comprising of lateral parts of levator ani muscles (Structure 9).

On T2W images, seminal vesicles appear as high-intensity multicystic structures and can be easily differentiated from significantly lower-signal intensity of periprostatic fat. On T1W images, this signal pattern is reversed, and seminal vesicles have lower signal intensity compared to periprostatic fat (Structures 3 and 4).

### 6.1. Prostatic Arteries

Proper identification of arteries and characterization of their anatomical variants (branches of internal iliac artery, accessory pudendal arteries, and number and origin of prostatic pedicles) is essential in patients qualified for prostatic arterial embolisation [2]. The anterior-lateral prostatic pedicle which provides blood mainly to the cranial pole and the central zone of the gland and posterior-lateral prostatic pedicle providing blood to the caudal pole and peripheral zone of the gland are the main object of interest. Identification of accessory or aberrant pudendal arteries responsible for arterial blood supply to corpora cavernosa is essential for their preservation during prostatectomy and may avoid erectile dysfunction caused by penile arterial insufficiency. DSA and CTA are considered to be a method of choice in evaluation of these vessels due to highest temporal resolution. Nonetheless, multiparametric MRI including DCE phase allows for proper recognition of PAs.

In most cases, internal pudendal artery (Structure 44, long blue arrow) and inferior gluteal artery (Structure 42, short blue arrow) are given off as e common gluteal-pudendal trunk (Structure 43, red arrow).

**Figure 9:**
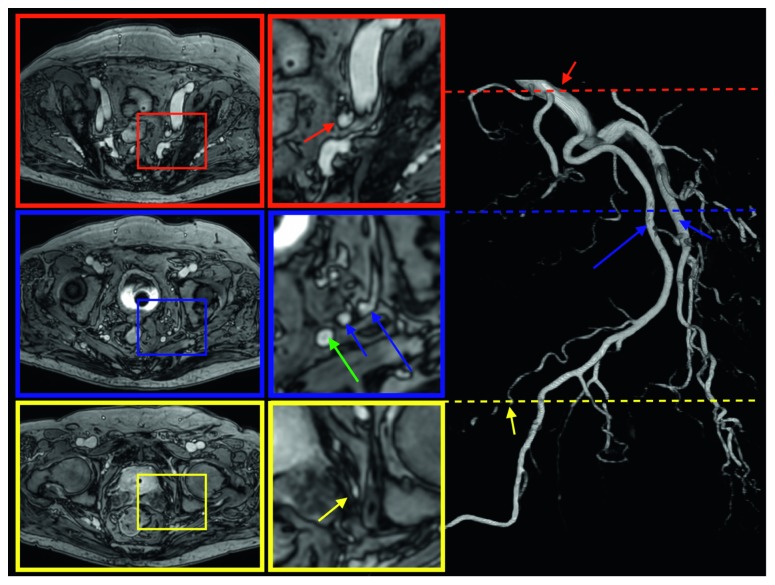
mDIXON postcontrast axial image of the prostate gland and corresponding angio-CT of arterial prostatic supply.

The prostatic arteries (PAs) (Structure 41, yellow arrow) supplying the prostate are usually small branches of the inferior vesical arteries (branches of the internal iliac artery (Structure 40)), internal pudendal artery (Structure 44, long blue arrow), and to a lesser extent inferior rectal artery (Figure 9). They run together with veins and nerves of prostatic plexus (Structure 7) forming neurovascular bundles (Structure 11) and course anteromedially entering prostate bilaterally (Figures 1 and 4). They are visualized in the rectoprostatic angle at the 5 o'clock and 7 o'clock positions. Apart from that, the middle rectal artery which is the more distant branch of internal iliac artery (Structure 40, green arrow) releases small prostate branches before it enters rectum. The inferior gluteal artery (Structure 42, short blue arrow) is seen as a branch of anterior division of the internal iliac artery which supplies the pelvic and gluteal muscles (Figure 9).

### 6.2. Prostatic Veins

The prostatic plexus, known also as Santorini's plexus (dorsal vein complex) (Structure 7), is located ventrally and laterally to the prostate and the external urethral sphincter (Structure 15) [5, 6]. It is covered by visceral endopelvic fascia (VEF) (Structure 31) and the detrusor apron (Structure 3), but both sphincter's fascia and VEF can be poorly appreciated on MR images. It drains blood from penile veins which penetrate fascia and drain to prostatic plexus. Posteriorly, it communicates with veins from rectoprostatic space, rectum, and seminal vesicles.

Prostatic venous plexus drains to paravertebral venous plexus (Batson's plexus) and to inferior vesical veins and further to the internal iliac vein, which comprises common path of metastasis of the prostate cancer. The communication with Batson's plexus is responsible for vertebral and intracranial metastatic spread of the prostatic cancer (Structure 5).

### 6.3. Lymph Nodes

**Figure 10:**
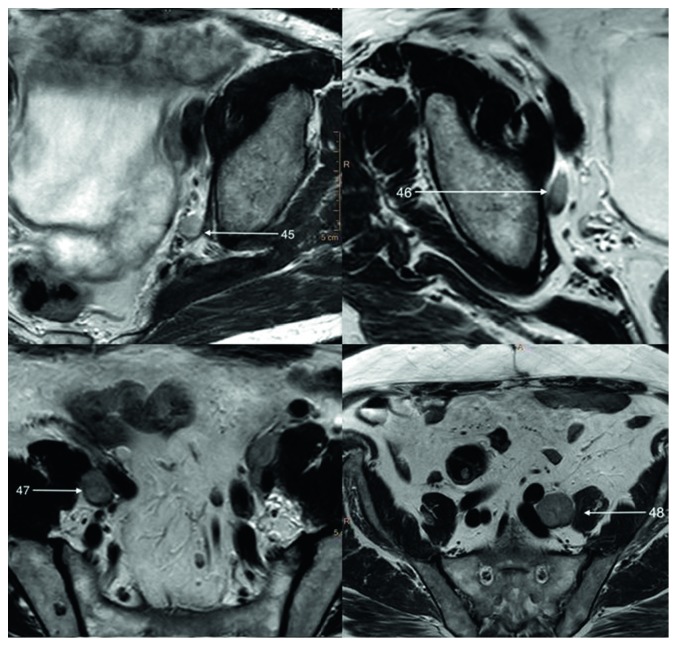
T2-weighted axial image of enlarged regional prostatic lymph nodes.

The lymphatic drainage of the prostate primarily drains to the internal iliac (Structure 45) and/or specifically the obturator channels (Structure 46) [5]. The other pathways of spread aim at external iliac (Structure 47), common iliac (Structure 48), and presacral and para-aortic lymph nodes (Figure 10).

Lymph vessels are located along with venous plexuses and are poorly visible on MR scans.

From the posterior surface of the prostate, lymph vessels drain together with lymph vessels of the bladder to posterior, lateral, and anterior lymph nodes of the bladder and to a lesser extent to the external iliac lymph nodes.

Seminal vesicles lymphatic drainage terminates both in external and internal iliac lymph nodes.

### 6.4. Nerve Fibers

Although distal nerve branches of pelvic cavity cannot be directly visualized in MRI in most cases, knowledge of their topography is essential in planning of nerve-sparing prostate surgery which in turn allows to retain sexual function or urinary continence [6]. On the other hand, understanding of nerve supply together with patient's symptoms enables us to interpret infiltration or impingement of nerve structures by pathologic masses.

Motor, secretory (autonomic), and sensory nerve fibers of prostate derive from prostatic plexus (Structure 7), which is continuation of the lower part of the inferior hypogastric plexus.

Inferior hypogastric plexus is formed by two (right and left) superior hypogastric nerves and is situated extraperitoneally at the bottom of lesser pelvis, just above levator ani muscle (Structure 9), on the both sides of rectum and urinary bladder. Laterally internal iliac vessels (Structure 40) and their branches are located. It creates broad, sagittally oriented plate with spreads from sacrum to bladder comprising ureter (Structure 37) anteriorly. Right and left hypogastric plexus are connected posteriorly. Inferior hypogastric plexus sends multiple branches which in turn form secondary plexuses-inferior hypogastric, middle rectal, inferior rectal, vesical, deferential, prostatic, and cavernous plexuses.

Medial and inferior rectal plexuses are paired structures, which connect with superior rectal plexus and supply medial and inferior portion of the rectum.

Vesical plexus lies bilaterally on urinary bladder (Structure 36) and gives off superior vesical nerves to the superior part of urinary bladder and inferior vesical nerves to the neck of urinary bladder, internal urethral sphincter, seminal vesicles, and beginning of urethra. Vesical plexus comprises also parasympathetic nerves responsible for emptying of the bladder by relaxation of internal urethral sphincter and contraction of detrusor of bladder.

Deferential plexus accompanies vas deferens (Structure 20) from its ampulla to cauda of epididymis giving off multiple branches to vas deferens and seminal vesicles.

Prostatic plexus (Structure 8) encompasses mostly posterior and inferior surface of prostate gland and lies within the fascia of the prostate. Its nerve branches penetrate the gland.

Cavernous nerve plexus is elongation of prostatic plexus anteriorly and inferiorly; then, it penetrates urogenital diaphragm and comes out at dorsal aspect of root of penis where it merges with nerve fibers of dorsal nerve of the penis. Impotence after radical prostatectomy is mostly due to damage of very fragile structures of cavernous or prostatic plexuses.

Sacral plexus (Structure 50) is located between piriformis muscle and pelvic fascia, posteriorly to internal iliac vessels, ureter, and sigmoid colon. It is the biggest nerve plexus of the human body—it derives from anterior branches of L4-C0 and innervates lower extremity, perineum, and pelvis.

**Figure 11:**
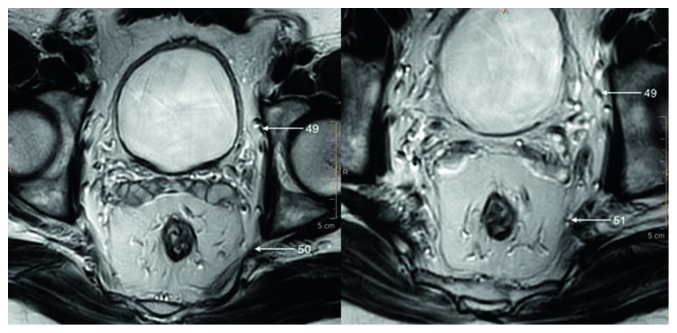
T2-weighted axial image of regional nerves.

Although pudendal nerve (Structure 51) does not supply prostate, it is the main nerve of perineum and plays crucial role in urinary continence and erectile function and therefore must be described in detail (Figure 11).

Pudendal nerve derives from sacral plexus, mainly from S3 and S4 nerves. It is different from other nerves of sacral plexus, because it carries not only sensory, motor, and sympathetic but also parasympathetic fibers. It is approximately 3 mm in diameter and can be discerned on T3 MRI. Pudendal nerve comes out of the lesser pelvis together with pudendal artery between the lower border of piriformis muscle and coccygeus muscle and then passes through the medial part of greater sciatic foramen. In gluteal region, it wraps around the posterior aspect of ischial spine and together with the internal pudendal artery and tendon of internal obturator muscle returns to the pelvis through lesser sciatic foramen and enters ischioanal fossa. Here, the nerve runs medially to ischial tuberosity and heads anteriorly in pudendal canal (Alcock's canal), which is formed by splitted obturator fascia. In posterior part of pudendal canal, the nerve divides into terminal branches—perineal nerve and dorsal nerve of the penis. Along its course, pudendal nerve gives off multiple branches. It innervates levator ani, coccygeus, external anal sphincter, bulbospongiosus, bulbocavernosus, ischiocavernosus, and transverse perineal and external urethral sphincter muscles. Sensory fibers carry sensation from skin of perineum and genitals apart from anterior portion of scrotum. Damage of pudendal nerve causes urinary incontinence, fecal incontinence, and impotence (Structure 6) [6].

## 7. Benign Prostatic Hyperplasia

Increased number (hyperplasia) of epithelial and stromal cells in the transition zone of the prostate is observed in most patients over 50 years [6]; therefore, it is considered by some sources to be a normal condition related to aging process.

BPH is seen on MR imaging as enlarged prostate with hyperplastic nodules in the transition zone and periurethral part of the central zone. It causes compression and displacement of the urethra which distorts anatomic landmarks. Hyperplastic nodules can have highly variable appearance. Differentiation between the PZ and the central gland is more clearly depicted than in normal, young prostate.

It is important to mention that intravesical prostate protrusion called a middle (or median) lobe is not meant to be taken into account while assessing the prostate volume.

Along with enlargement of prostate, prostatic arteries become more prominent as the gland demands stronger blood supply.

## 8. Physiology of the Prostate Gland Based on Spectroscopic Findings

Although the clou of the paper is to present the normal anatomy of the prostate gland, the spectroscopic imaging may be a part of multiparametric MR study. It provides information about levels of specific metabolites. If the normal prostate citrate (produced by normal epithelial cells) has peak at 2.6 ppm, choline (which is a precursor of phospholipids of the cellular membrane) has peak at 3.2 ppm and creatine (which helps to supply energy to the cells) has peak at 3 ppm [1, 6]. Increase of choline and decrease of citrate are a typical spectroscopic feature of prostate cancer. Apart from direct measurement, ratio of choline + creatine/citrate is usually being established. The ratio shows lower values in the periphery than in the central gland. The normal range is from 0.22 ± 0.013 up to 0.5. The ratios >0.5 are regarded as suspicious, >1 as very suspicious, and >2 as abnormal.

## 9. Conclusions

Introduced by McNeal in 1968, zonal anatomy of prostate constitutes the base of modern prostate imaging. Nowadays, with implementation of new sequences and high field systems, we are able to assess detailed prostate anatomy, thus enabling more precise diagnosis, better assessment of surrounding structures including lymph nodes and vessels. Very subtle structures as small nerve branches, although cannot be directly seen on imaging, can be assessed with implementation of knowledge of anatomical topography and relationship of structures. Further advances in technology give promising perspectives for imaging of the prostate and its surroundings.
